# A Diagnostic Challenge: Sclerosed Hepatic Haemangioma Mimicking Malignancy

**DOI:** 10.7759/cureus.94449

**Published:** 2025-10-13

**Authors:** Umar A Lakhani

**Affiliations:** 1 Gastroenterology, Royal Shrewsbury Hospital, Shrewsbury, GBR

**Keywords:** benign, heart failure, hepatic sclerosed hematoma, percutaneous liver biopsy, pulmonary embolism

## Abstract

A 75-year-old lady presented to Accident and Emergency (A&E) with acute worsening shortness of breath and new palpitations. On assessment, she was found to have fast atrial fibrillation, signs of heart failure, and bilateral peripheral oedema. Investigations revealed pulmonary embolism and an incidental hepatic lesion on CT imaging, suspicious for malignancy. Multidisciplinary discussion led to liver biopsy, which confirmed a benign sclerosed haemangioma. The patient was managed conservatively for cardiac and thromboembolic complications. This case highlights the difficulty in differentiating sclerosed haemangiomas from malignant liver lesions on imaging alone and underscores the importance of biopsy and multidisciplinary decision-making in elderly patients with comorbidities. Learning points include diagnostic vigilance, safe management of anticoagulation, and avoidance of unnecessary surgery.

## Introduction

Hepatic haemangiomas are the most common benign liver tumours, accounting for a prevalence of roughly 0.4-20% [1]; but the sclerosed (hyalinised) variant accounts for fewer than 2% of all cases [2]. These lesions develop as cavernous haemangiomas undergo fibrosis, hyalinisation, and vascular thrombosis. In light of these pathological changes, the typical radiological features of haemangiomas-peripheral nodular enhancement with progressive centripetal filling-are often absent. Instead, sclerosed haemangiomas may display irregular peripheral enhancement, central hypodensity, capsular retraction, or calcification, features that closely mimic intrahepatic cholangiocarcinoma or secondary metastases [3,4].

Misinterpretation of imaging can result in unnecessary surgical resections. Histological confirmation is therefore crucial in atypical cases. We present a case in which a large sclerosed hepatic haemangioma was discovered incidentally during investigation for acute cardiopulmonary symptoms. This case is notable for its diagnostic complexity, the impact of comorbidities such as heart failure and pulmonary embolism, and the pivotal role of multidisciplinary decision-making in avoiding major hepatic surgery.

## Case presentation

A 75-year-old woman presented with a seven-day history of progressive shortness of breath, palpitations, and ankle swelling. She denied chest pain or fever. On examination, she was found tachycardic with an irregular pulse at 132 beats per minute. Blood pressure was 118/76 mmHg, respiratory rate was 24 breaths per minute, and oxygen saturation was 94% on room air. Clinical examination revealed raised jugular venous pressure, bibasal crackles, and bilateral pitting oedema, consistent with decompensated heart failure.

Investigations

**Figure 1:**
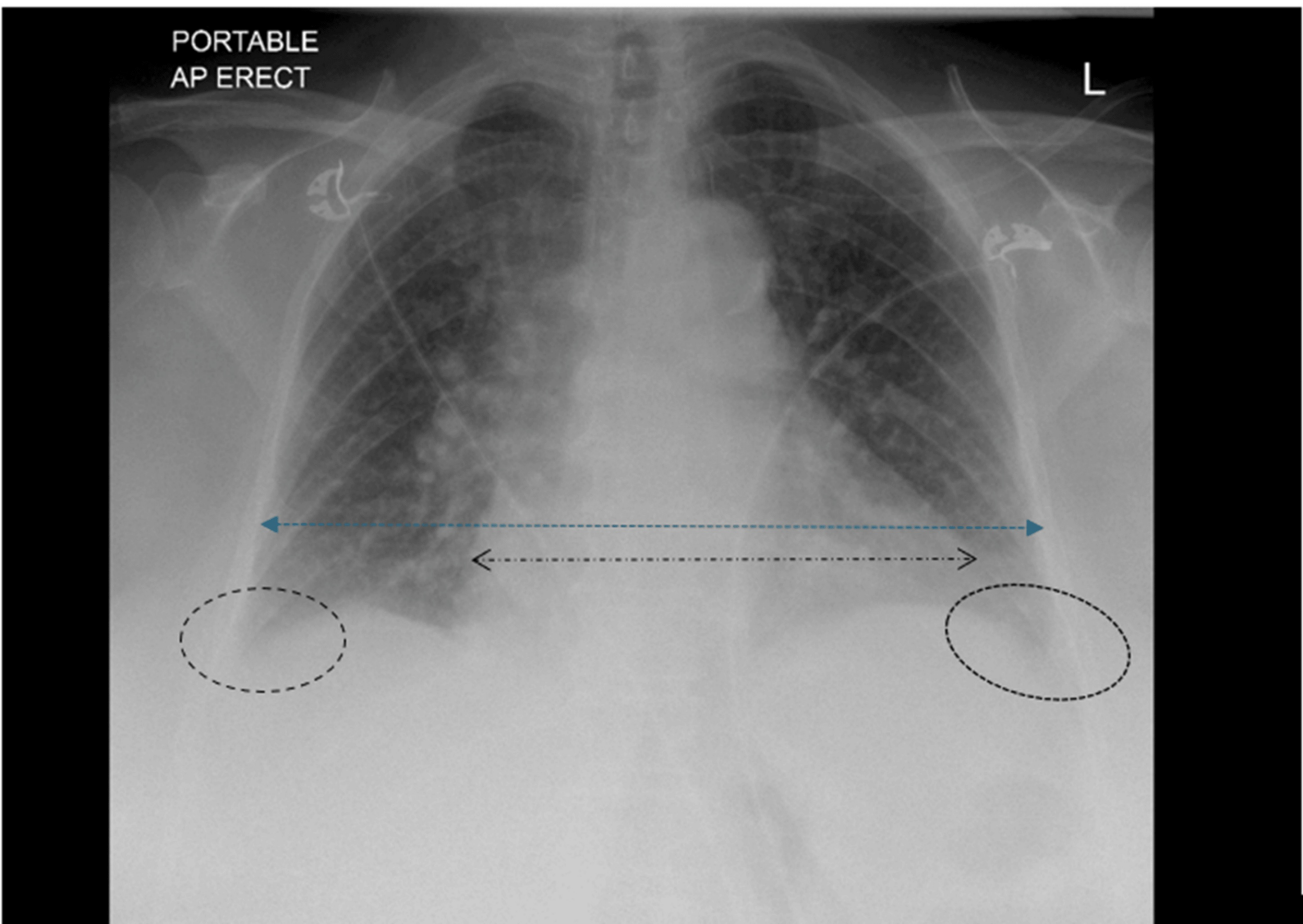
Chest X-ray In this image, the heart borders have been highlighted in comparison to the thoracic borders, which clearly demonstrates a cardiothoracic ratio of >0.5. The costophrenic angles are also blunted, which have been highlighted bilaterally. As can be seen in this patient's chest X-ray, an increased cardiothoracic ratio and blunting of the costophrenic angles are visible, suggesting heart failure with pleural effusion, potentially secondary to heart failure.

The electrocardiogram demonstrated fast atrial fibrillation with a ventricular rate of approximately 140 beats per minute. A chest radiograph revealed cardiomegaly and bilateral pleural effusions (Figure 1).

The patient's laboratory findings are summarised in Table 1.

**Table 1:** Initial laboratory investigations

Test	Result	Reference range
Haemoglobin	139 g/L	115–160 g/L
White cell count	9.6 × 10⁹/L	4.0–11.0 × 10⁹/L
C-reactive protein	40 mg/L	< 5 mg/L
B-type natriuretic peptide	1946 pg/mL	< 100 pg/mL
D-dimer	4109 µg/L	< 500 µg/L
International normalised ratio	1.1	0.9–1.2
Urea and electrolytes	Within normal limits	—
Carbohydrate antigen 19-9	15 U/mL	< 37 U/mL

**Figure 2:**
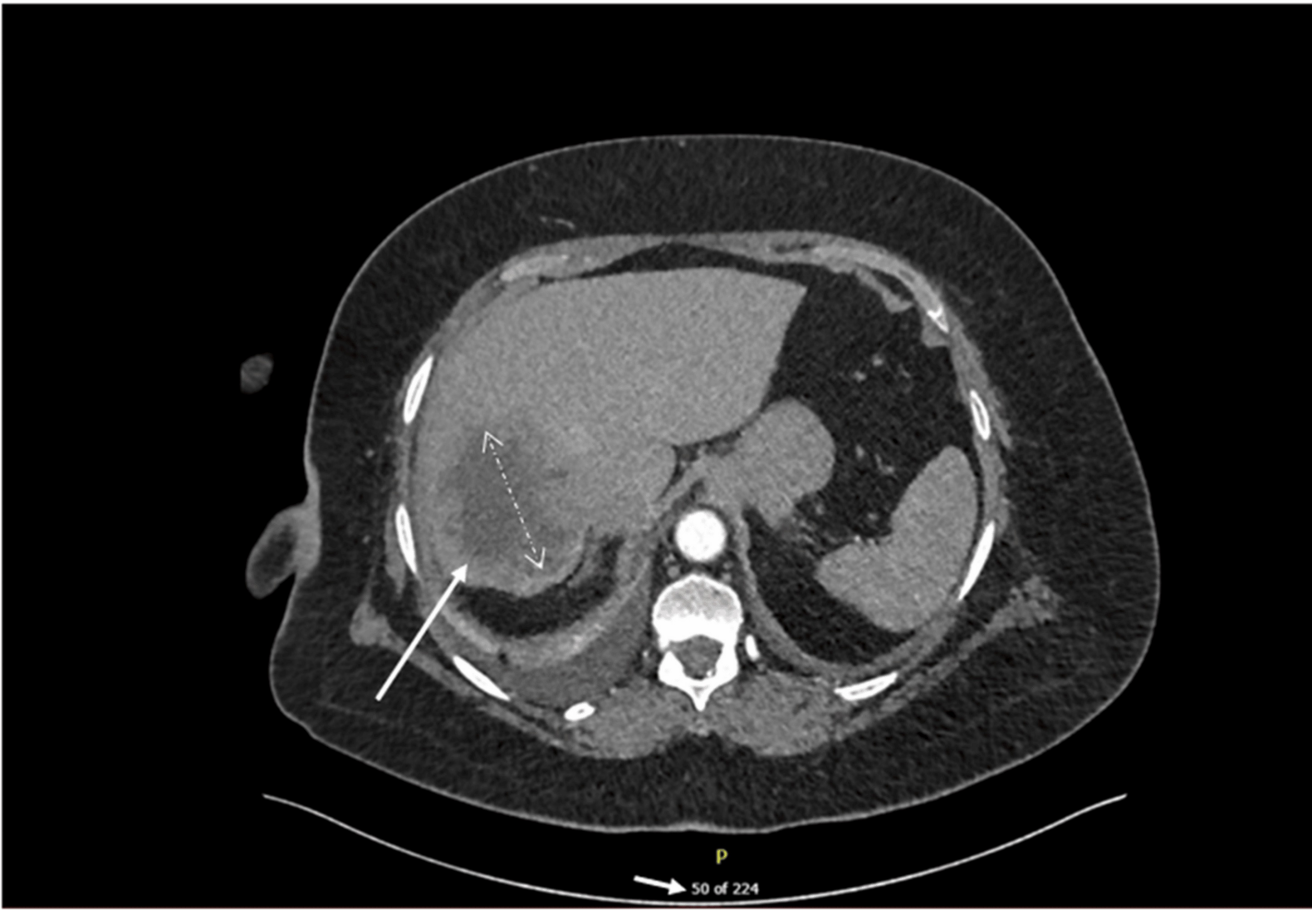
CTPA image showing hepatic lesion CTPA: Computed tomography pulmonary angiography There is a large hepatic dome lesion (white arrow) measuring about 8 cm in diameter, showing peripheral irregular enhancement with central hypodensity. There are multiple flecks of calcifications within the lesion.

Computed tomography pulmonary angiography confirmed right-sided pulmonary emboli. Incidentally, an 8 cm lesion was identified in the dome of the liver. It demonstrated irregular peripheral enhancement, central hypodensity, and calcifications, raising strong suspicion for primary or secondary malignancy (Figure 2).

**Figure 3:**
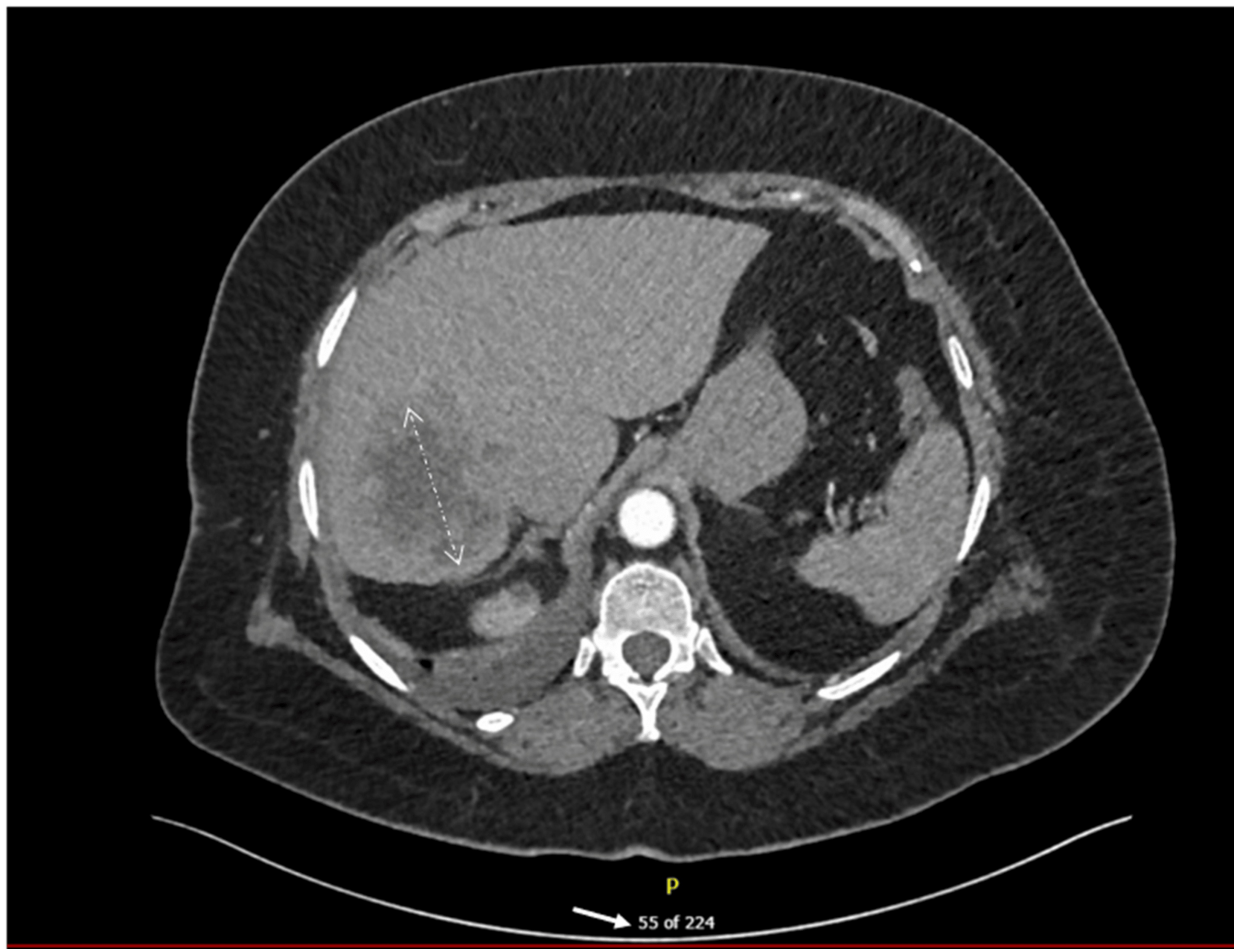
CTPA image showing extent of lesion CTPA: Computed tomography pulmonary angiography Bilateral hepatic cysts, measuring 12 mm on the right, with a left interpolar one at 2 cm.

A dedicated triple-phase computed tomography of the liver confirmed the lesion, showed two small simple hepatic cysts, but revealed no lymphadenopathy or extrahepatic disease (Figure 3).

Differential diagnosis

The differential diagnosis included intrahepatic cholangiocarcinoma, hepatocellular carcinoma, metastatic deposits, and sclerosed haemangioma.

Management and clinical course

**Figure 4:**
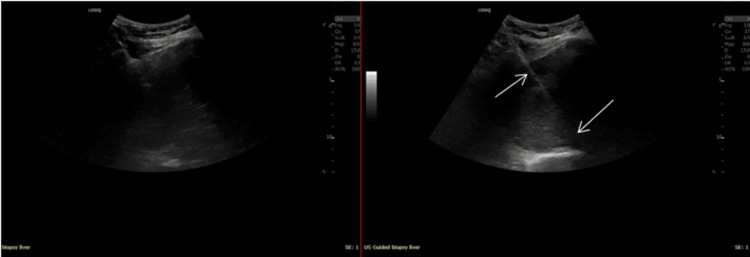
Ultrasound-guided hepatic biopsy images This is an image captured during the ultrasound-guided biopsy process, showing the needle (white arrows) used in an intercostal approach.

The patient was treated for acute heart failure and atrial fibrillation with intravenous furosemide, bisoprolol, and digoxin. Anticoagulation with apixaban was initiated for pulmonary embolism. Given the suspicious hepatic lesion, the case was reviewed by both the upper gastrointestinal and hepatobiliary multidisciplinary teams. A percutaneous liver biopsy was performed. Histology demonstrated dense fibrous hyalinised stroma with collapsed vascular channels lined by bland endothelial cells, with no atypia or malignancy. The diagnosis of sclerosed haemangioma was made (Figure 4).

Despite this, persistent radiological suspicion as well as a "technically difficult" first biopsy prompted a repeat biopsy at a tertiary centre, which confirmed the same benign pathology. The patient remained asymptomatic from a hepatic perspective, with improved weight and appetite, and was managed conservatively with imaging surveillance.

## Discussion

Sclerosed haemangiomas represent a rare but important diagnostic pitfall. In contrast to cavernous haemangiomas, which demonstrate the classic pattern of peripheral nodular enhancement and centripetal fill-in, sclerosed haemangiomas often lack these features. Instead, they present with irregular peripheral enhancement, capsular retraction, and calcification, which mimic malignant lesions [2-4].

Several published case reports and reviews highlight that misdiagnosis is a common occurrence. Lai et al. reported multiple cases where sclerosed haemangiomas were mistaken for intrahepatic cholangiocarcinoma, leading to unnecessary resections [5]. Tanaka et al. described a series in which up to 30% of patients undergoing hepatic resection for presumed malignancy were ultimately diagnosed with benign sclerosed haemangiomas [6]. Our case parallels these findings but adds further complexity given the patient’s acute cardiopulmonary comorbidities and the risks of biopsy under anticoagulation.

Histopathology remains the definitive diagnostic tool. Characteristic findings include fibrous stroma, hyalinisation, and collapsed vascular channels lined by endothelial cells without atypia. In equivocal cases, repeat biopsy may be warranted to ensure diagnostic certainty and avoid resection of benign lesions. Our patient’s case underscores three key points. First, imaging alone cannot reliably distinguish sclerosed haemangiomas from malignancy. Second, in elderly patients with significant comorbidities, histological confirmation is critical to prevent unnecessary major hepatic surgery. Third, a multidisciplinary review was vital in balancing the competing risks of anticoagulation, biopsy, and surgical candidacy.

## Conclusions

Sclerosed hepatic haemangiomas are rare benign lesions that frequently mimic hepatic malignancies on imaging. Misdiagnosis can lead to unnecessary hepatic resections. Histological confirmation is essential, particularly in elderly or comorbid patients, where surgery carries a significant risk. This case demonstrates how a multidisciplinary approach, incorporating careful biopsy and repeat histological confirmation, enabled an accurate diagnosis and safe conservative management.
